# Supplementary material for: Microsecond fingerprint stimulated Raman spectroscopic imaging by ultrafast tuning and spatial-spectral learning
Source: Nat Commun. 2021 May 24;12:3052. doi: 10.1038/s41467-021-23202-z (PMC8144602; doi:10.1038/s41467-021-23202-z)
Supplement: Supplementary file 1 — Supplementary Information [file 41467_2021_23202_MOESM1_ESM.pdf]

Supplementary information

Microsecond Fingerprint Stimulated Raman Spectroscopic Imaging by Ultrafast Tuning and Spatial-Spectral Learning

Haonan Lin et al.

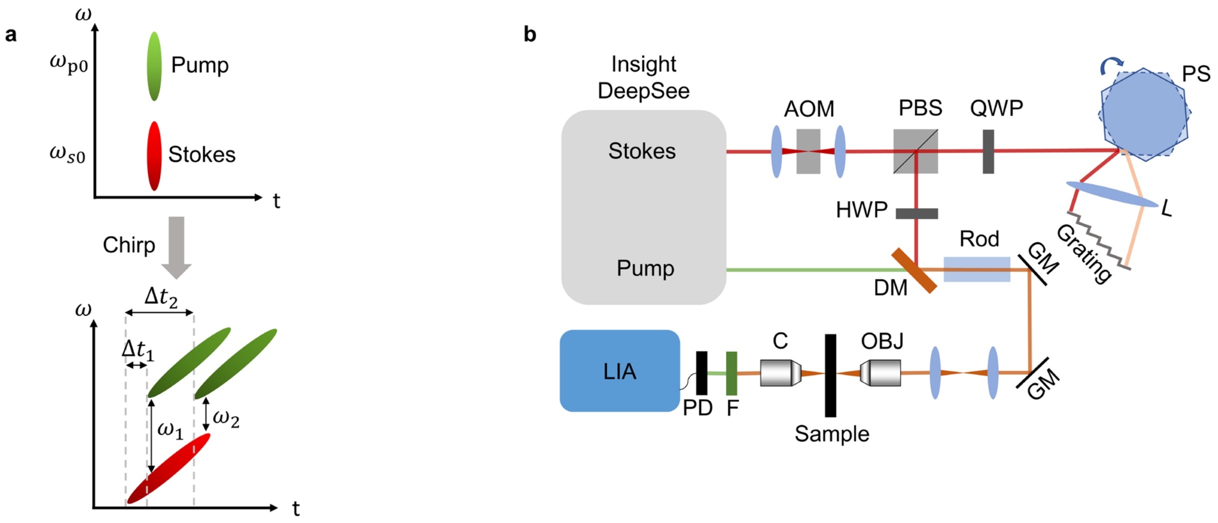

Supplementary Figure 1. Optical setup of the ultrafast tuning SRS system. (a) Concept of spectral focusing. (b) Optical setup. AOM, acousto-optic modulator; C, condenser; F, filter; GM, galvo mirror; HWP, half-wave plate; L, lens; LIA, lock-in amplifier; OBJ, objective; PBS, polarizing beam splitter; PD, photodiode; PS, polygon scanner; QWP, quarter-wave plate.

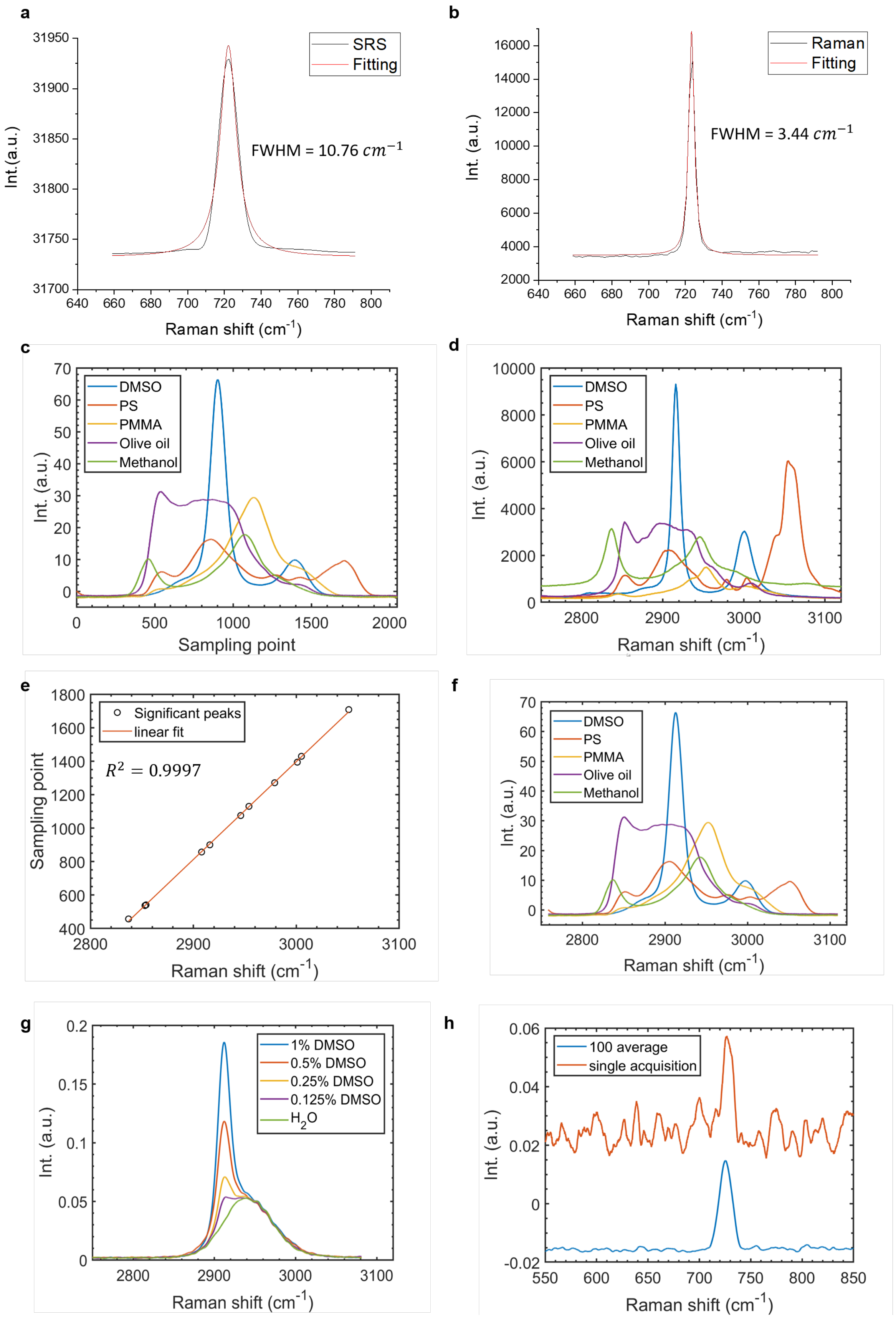
a
SRS
Fitting
FWHM = 10.76 cm^{-1}
Int.(a.u.)
Raman shift (cm-1)
b
Raman
Fitting
FWHM = 3.44 cm^{-1}
Int. (a.u.)
Raman shift (cm-1)
c
DMSO
PS
PMMA
Olive oil
Methanol
Int. (a.u.)
Sampling point
d
DMSO
PS
PMMA
Olive oil
Methanol
Int. (a.u.)
Raman shift (cm-1)
e
Significant peaks
linear fit
$R^2 = 0.9997$
Sampling point
Raman shift (cm-1)
f
DMSO
PS
PMMA
Olive oil
Methanol
Int. (a.u.)
Raman shift (cm-1)
g
1% DMSO
0.5% DMSO
0.25% DMSO
0.125% DMSO
H_2O
Int. (a.u.)
Raman shift (cm-1)
h
100 average
single acquisition
Int. (a.u.)
Raman shift (cm-1)

Supplementary Figure 2. The spectral resolution, spectral linearity and sensitivity of ultrafast delay-line tuning SRS. (a) SRS spectrum of adenine and Lorenz one-peak fitting. (b) Spontaneous Raman spectrum of adenine and Lorenz one-peak fitting. (c) Raw SRS spectra (from digitizer) of DMSO, PS, PMMA, olive oil and methanol obtained by polygon delay-line scanning system. Sampling point refers to the number of sampled data points after a trigger, which corresponds to acquisition time from the sampling trigger. (d) Spontaneous Raman spectra of the same chemicals. (e) Mapping of Raman shifts to sampling point number of the digitizer. (f) SRS spectra from (c) after wavenumber calibration. (g) SRS spectra of water and DMSO solutions with different concentrations. (h) Fingerprint SRS spectra of adenine by single acquisition and by 100 averaging, baseline manually offset for display. Int., Intensity. a.u., arbitrary unit.

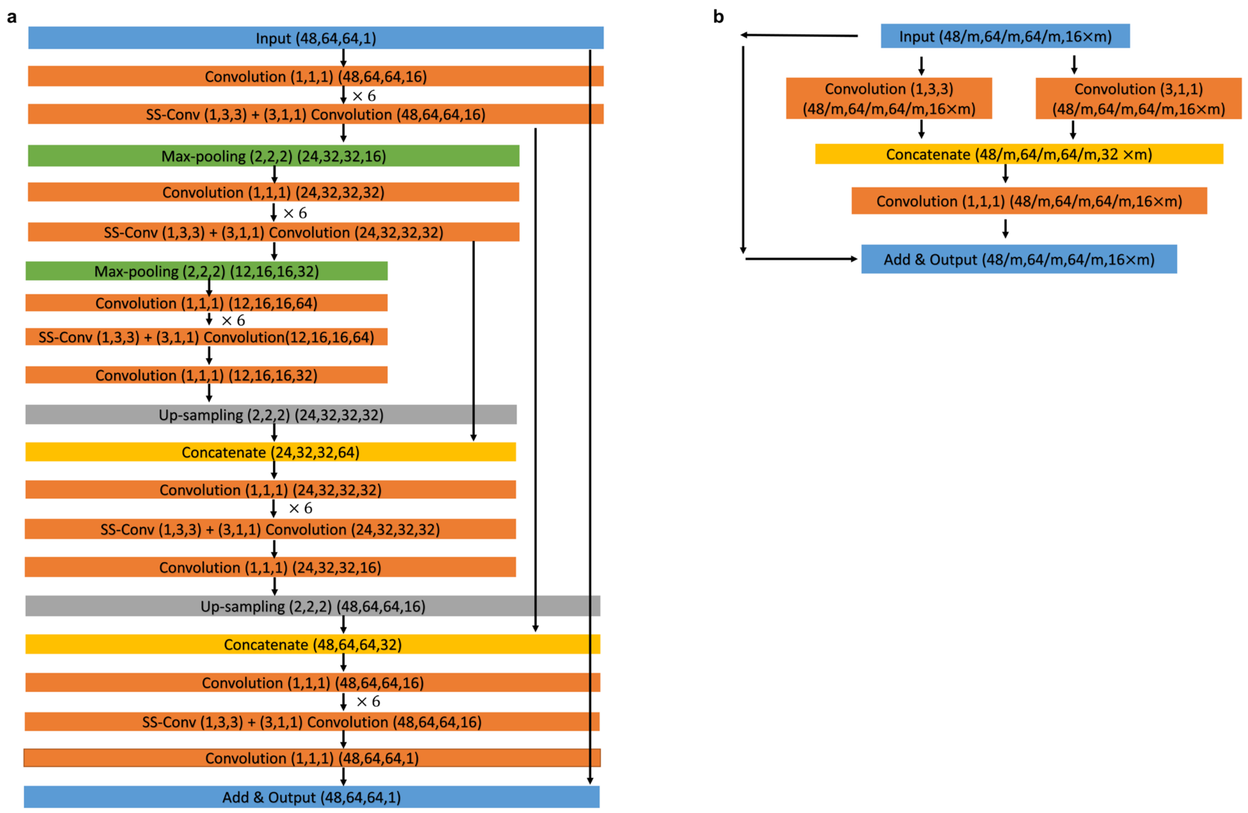

Supplementary Figure 3. **SS-ResNet structure for SNR recovery of spectroscopic images**. (a) The complete structure of the network. Orange layers represent either conventional or spatial-spectral convolution. Green layers represent max-pooling layers with (2,2,2) kernel size. Gray layers stand for (2,2,2) up-sampling while the yellow layers concatenate up-sampled feature maps with corresponding feature maps in the previous encoder level. (b) Structure of each SS-Conv layer. The input is separately filtered spatially and spectrally through a (1,3,3) and (3,1,1) convolution. The two outputs are concatenated and passed through a (1,1,1) convolution to reduce the feature channels. Adding with the input yields the final output of the convolution layer.

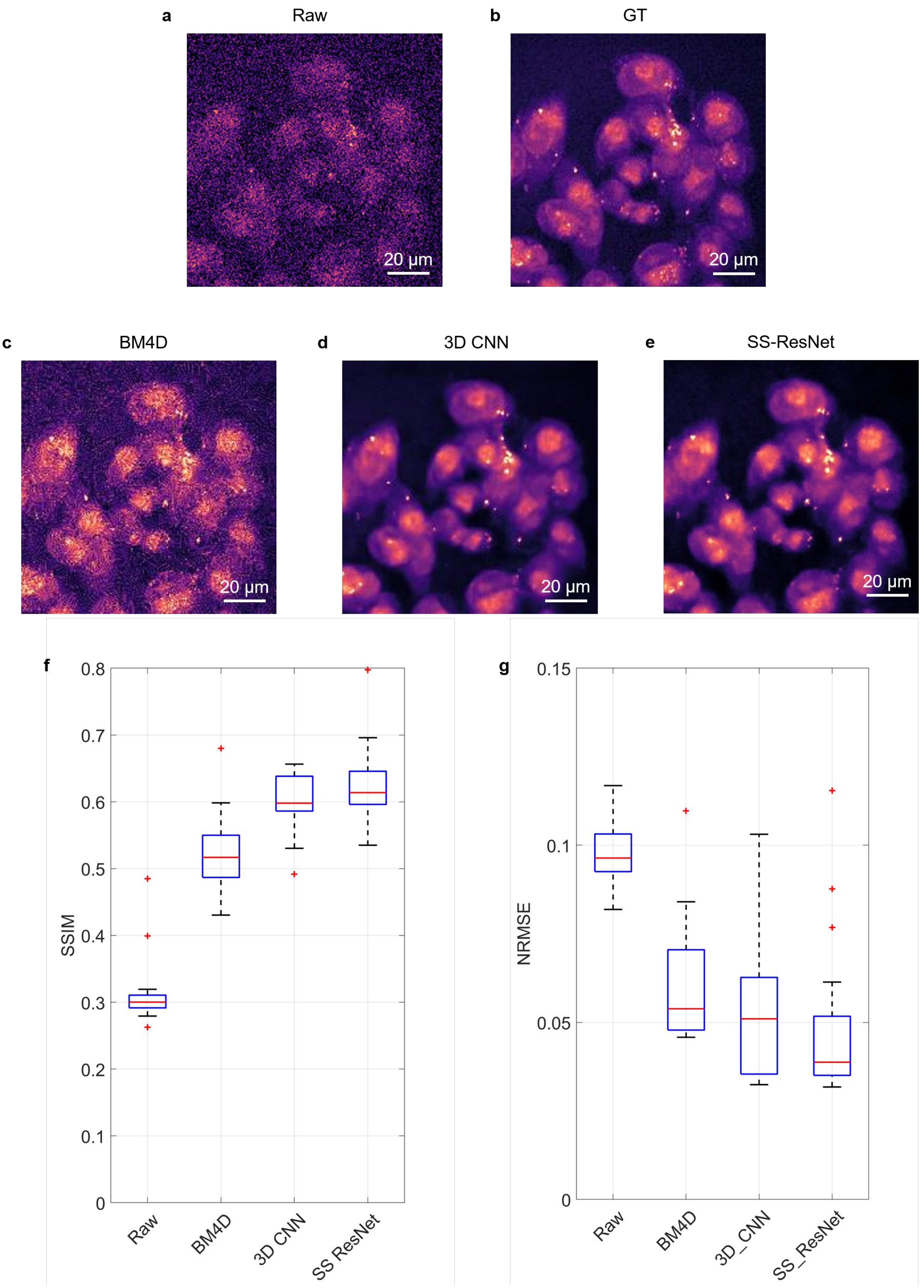

a
Raw
20 µm
b
GT
20 µm
c
BM4D
20 µm
d
3D CNN
20 µm
e
SS-ResNet
20 µm
f
SSIM
0.8
0.7
0.6
0.5
0.4
0.3
0.2
0.1
0
Raw
BM4D
3D CNN
SS ResNet
g
NRMSE
0.15
0.1
0.05
0
Raw
BM4D
3D_CNN
SS_ResNet

Supplementary Figure 4. Comparing SS-ResNet with 3D CNN and BM4D for the MiaPaCa-2 dataset. (a) Raw single acquisition data. (b) High-SNR ground truth (GT) data by averaging the same FOV 100 times. (c) BM4D denoising from the raw image. (d) 3D CNN recovery from the raw image. (e) SS-ResNet recovery from the raw image. (f-g) Quantitation of prediction error for fixed Mia PaCa-2 validation dataset. Box plots (n=19) show NRMSE (lower is better) and SSIM (higher is better) for the raw, BM4D, 3D CNN and SS-ResNet recovery. The boxes show interquartile range (IQR), the red line indicates medians, the black lines represent whiskers which extend to 1.5 times of the IQR, the red datapoints are outliers exceeding the whiskers. NRMSE, normalized root mean square error. SSIM, structural similarity index.

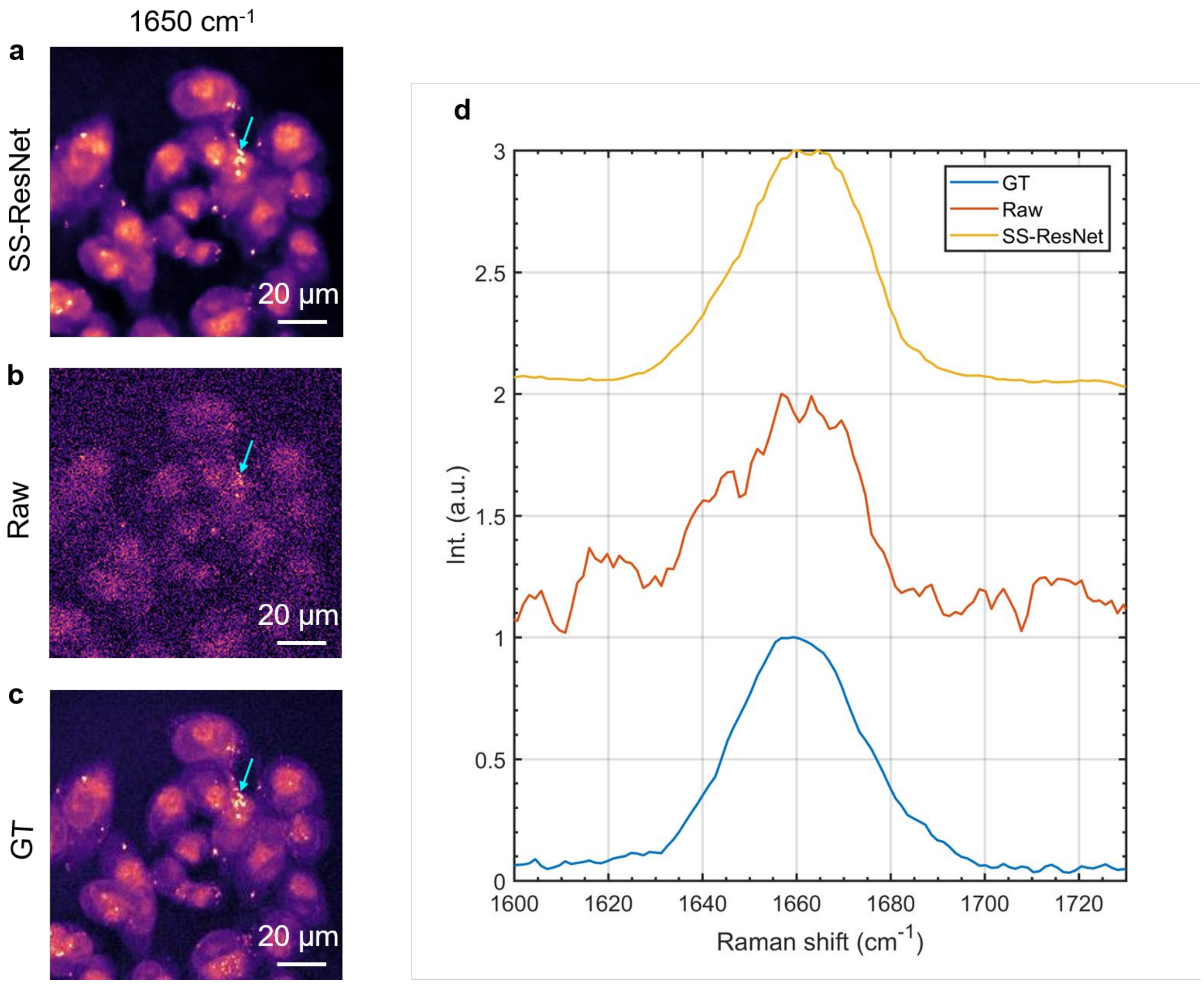

Supplementary Figure 5. Spectral profile analysis for the MiaPaCa-2 dataset. (a) SS-ResNet recovery from the raw image. (b) Single acquisition raw data. (c) High-SNR ground truth (GT) data by averaging the same FOV 100 times. The testing set contains n=19 independent image pairs that are not included during network training. (d) Spectral profiles from the same pixel (marked in (a)-(c)) for Raw, GT and SS-ResNet recovery. Profiles for SS-ResNet and Raw are manually shifted for display. Int., Intensity. a.u., arbitrary unit.

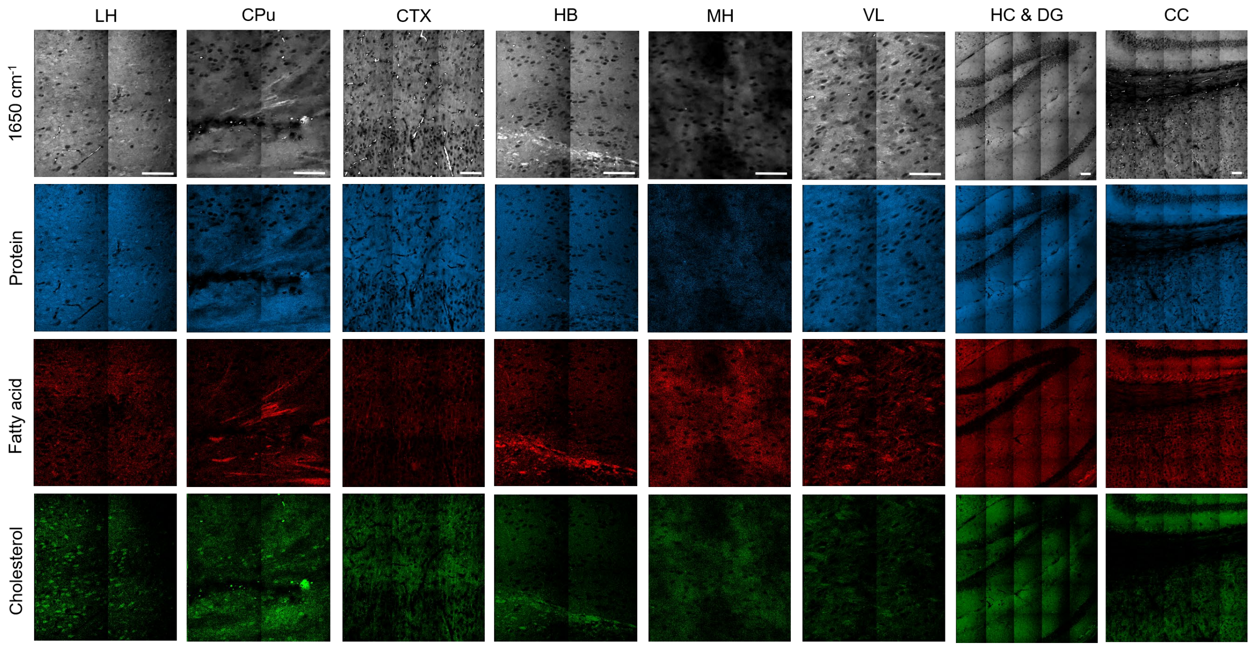

Supplementary Figure 6. **Whole brain training & validation set**. Ground truth (GT) by 100 averaging. SRS image at 1650 cm^{-1} and chemical maps by spectral unmixing of the spectroscopic image stack are shown for each region. Each region was stitched by small images ranging from 2 × 2 to up to 5 × 5. A total of n=50 small images were used for training. Scale bars, 50 µm.

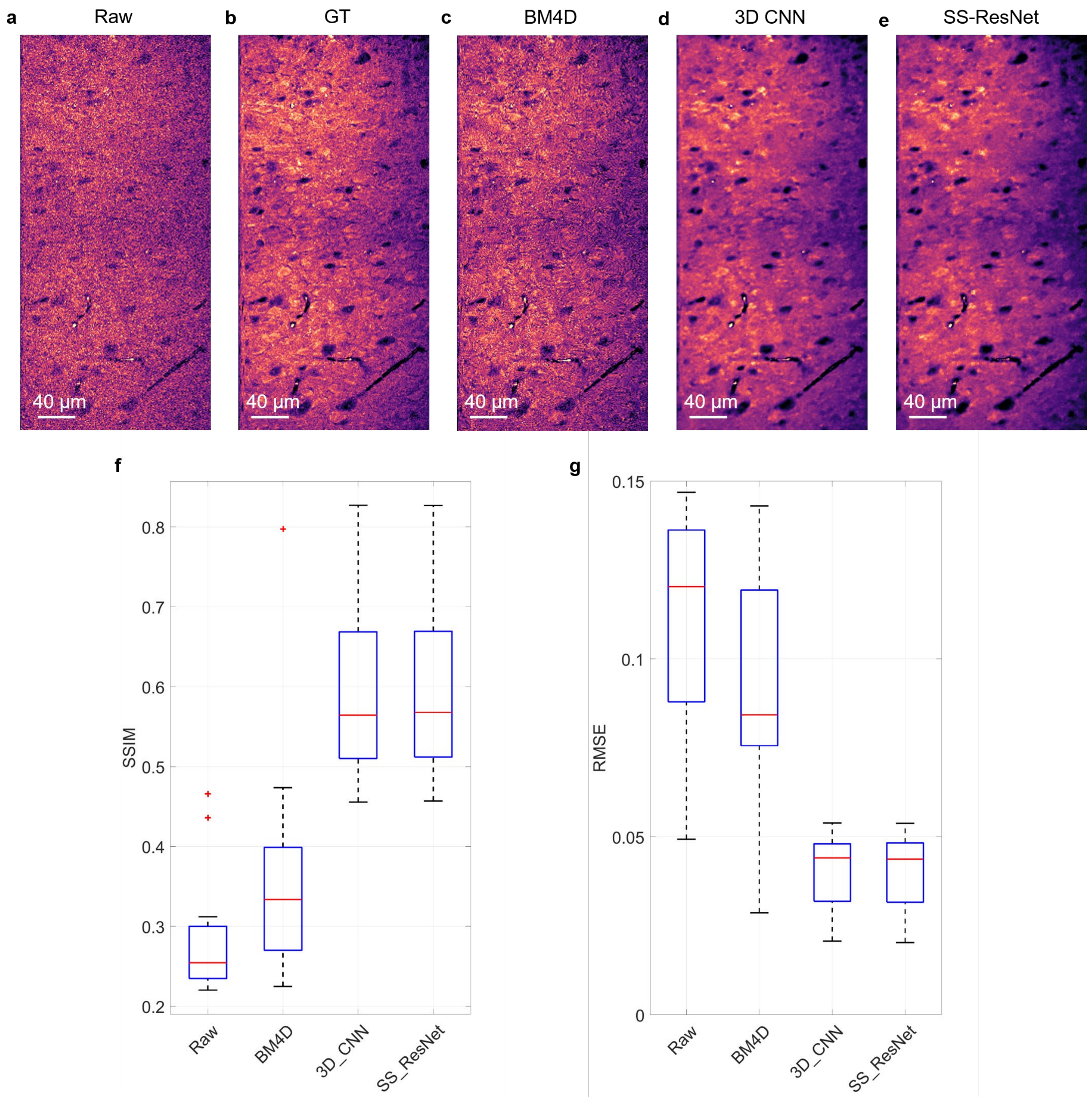

Supplementary Figure 7. Comparing SS-ResNet with 3D CNN and BM4D for the brain dataset. (a) Raw single acquisition data. (b) High-SNR ground truth (GT) data by averaging the same FOV 100 times. (c) BM4D denoising from the raw image. (d) 3D CNN recovery from the raw image. (e) SS-ResNet recovery from the raw image. (f-g) Quantitation of prediction error for brain dataset, including 15 spectroscopic images. Box plots (n=15) show RMSE (lower is better) and SSIM (higher is better) for the raw, BM4D, 3D CNN and SS-ResNet recovery. The boxes show interquartile range (IQR), the red line indicates medians, the black lines represent whiskers which extend to 1.5 times of the IQR, the red datapoints are outliers exceeding the whiskers. NRMSE, normalized root mean square error. SSIM, structural similarity index.

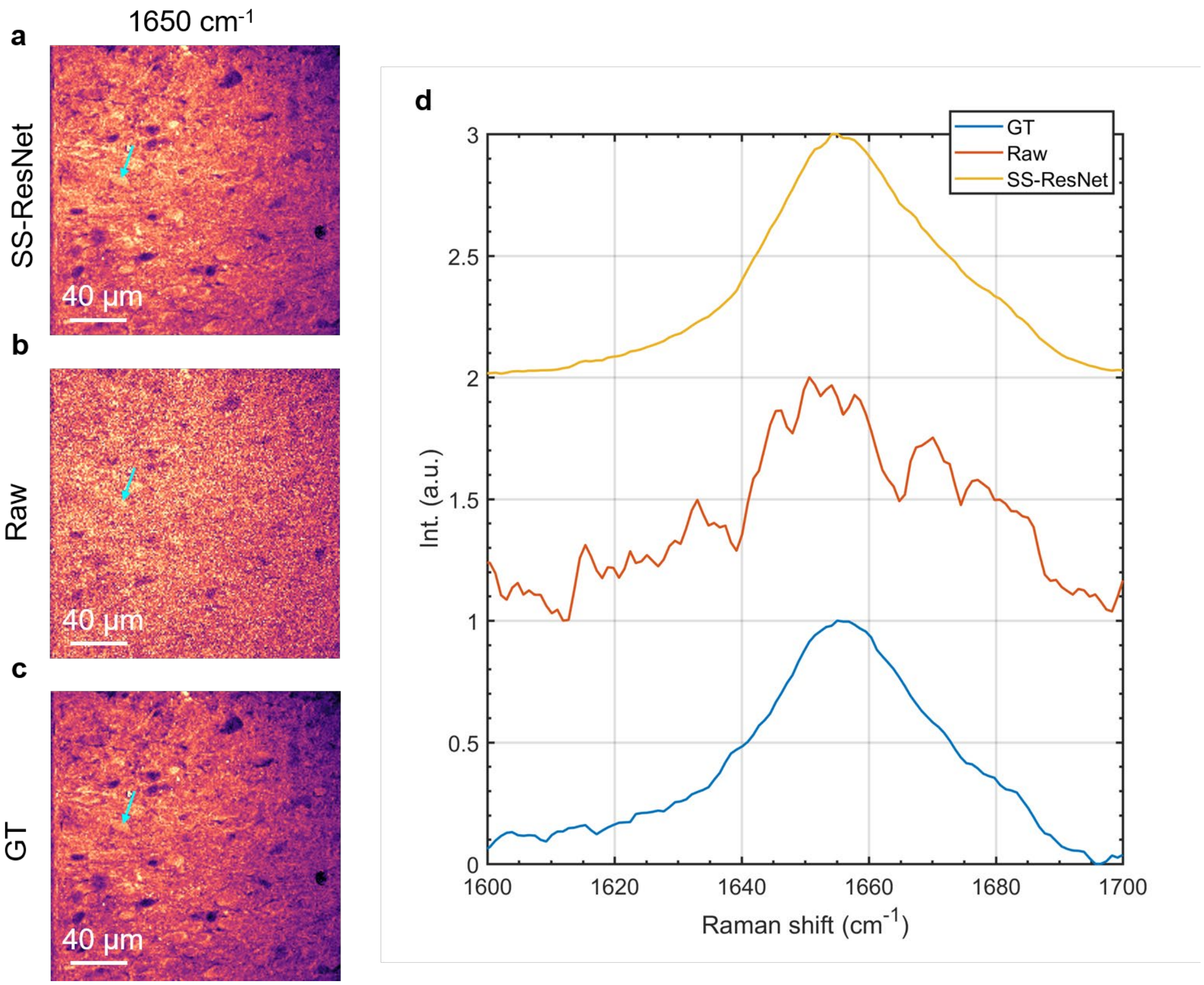

Supplementary Figure 8. Spectral profile analysis for the brain dataset. (a) SS-ResNet recovery from the raw image. (b) Single acquisition raw data. (c) High-SNR ground truth (GT) data by averaging the same FOV 100 times. The testing set contains n=15 independent image pairs that are not included during network training. (d) Spectral profiles from the same pixel (marked in (a)-(c)) for Raw, GT and SS-ResNet recovery. Profiles for SS-ResNet and Raw are manually shifted for display. Int., Intensity. a.u., arbitrary unit.

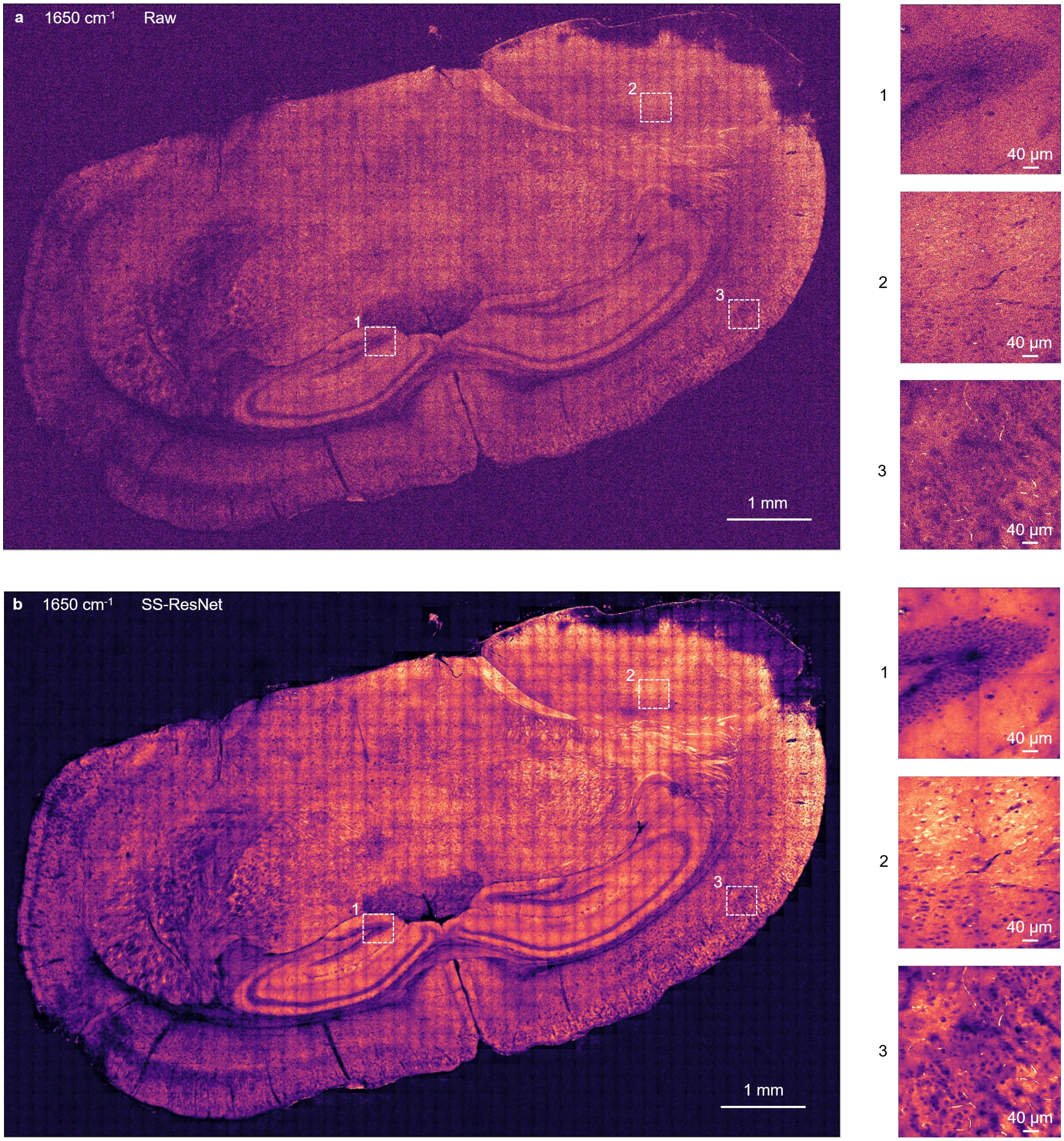

Supplementary Figure 9. **Mouse whole brain SRS image at 1650 cm^{-1}**. (a) Raw image. (b) SS-ResNet recovery from the raw image.

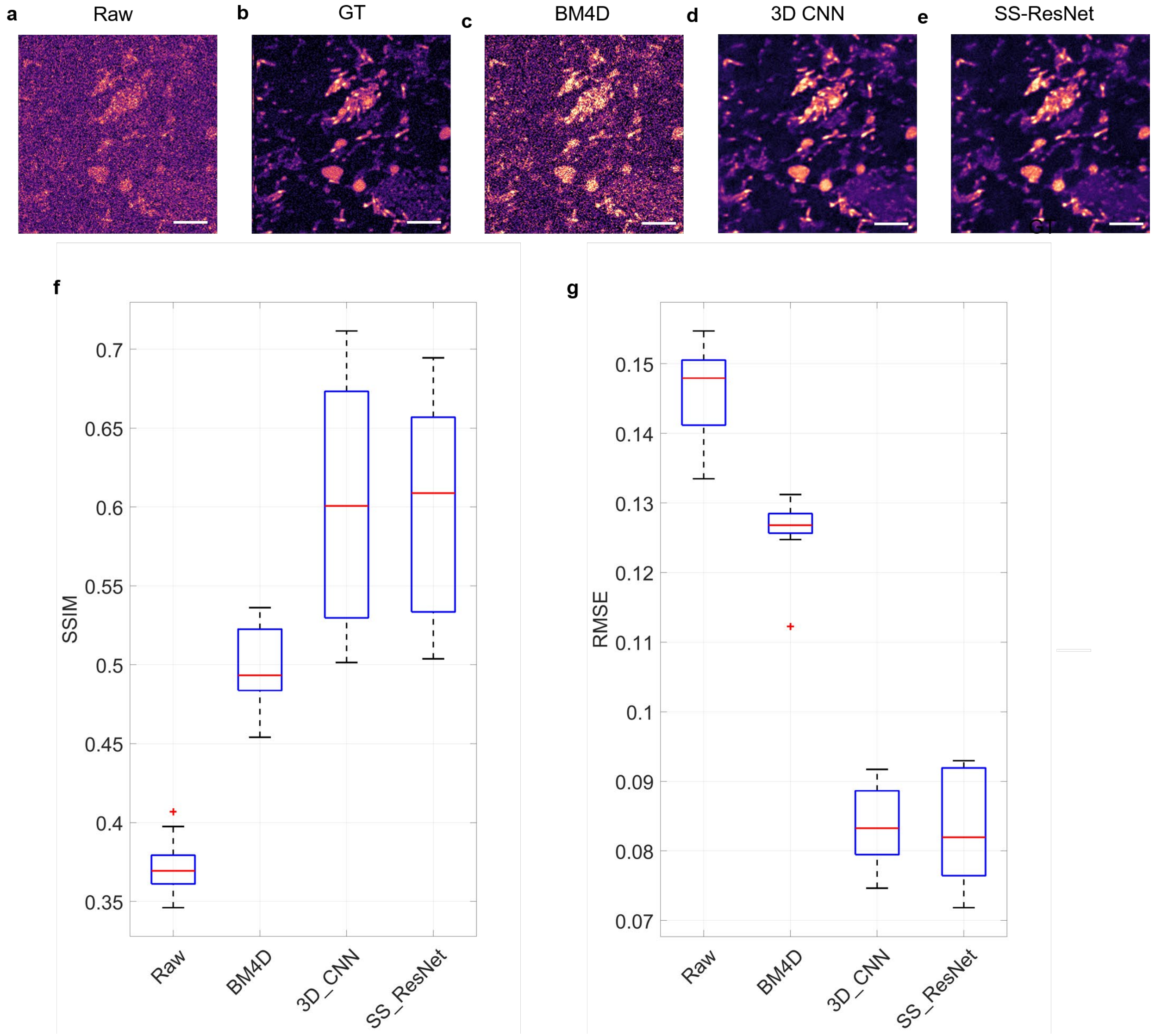

Supplementary Figure 10. Comparing SS-ResNet with 3D CNN and BM4D for the *E. coli* dataset. (a) Raw single acquisition data. (b) High-SNR GT data by averaging the same FOV 100 times. (c) BM4D denoising from raw image. (d) 3D CNN recovery from the raw image. (e) SS-ResNet recovery from the raw image. (f-g) Quantitation of prediction error for the brain dataset with n=13 independent spectroscopic SRS images. Box plots (n=13) show RMSE (lower is better) and SSIM (higher is better) for the raw, BM4D, 3D CNN and SS-ResNet recovery. The boxes show interquartile range (IQR), the red line indicates medians, the black lines represent whiskers which extend to 1.5 times of the IQR, the red datapoints are outliers exceeding the whiskers. NRMSE, normalized root mean square error. SSIM, structural similarity index. Scale bars, 10 μm.

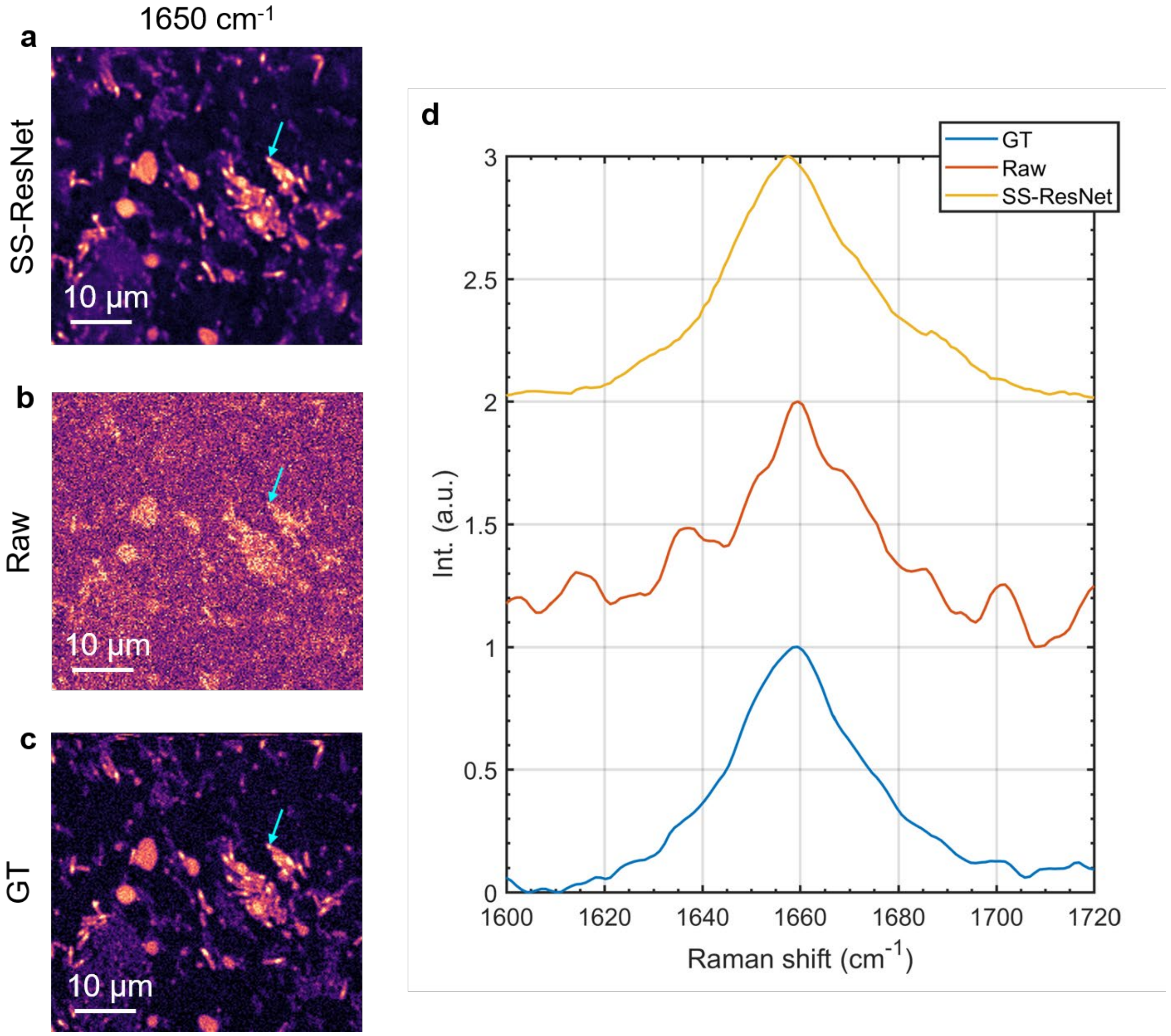

Supplementary Figure 11. Spectral profile analysis for the *E. coli* dataset. (a) SS-ResNet recovery from the raw image. (b) Single acquisition raw data. (c) High-SNR ground truth (GT) data by averaging the same FOV 100 times. The testing set contains n=13 independent image pairs that are not included during network training. (d) Spectral profiles from the same pixel (marked in (a)-(c)) for Raw, GT and SS-ResNet recovery. Profiles for SS-ResNet and Raw are manually shifted for display. Int., Intensity. a.u., arbitrary unit.

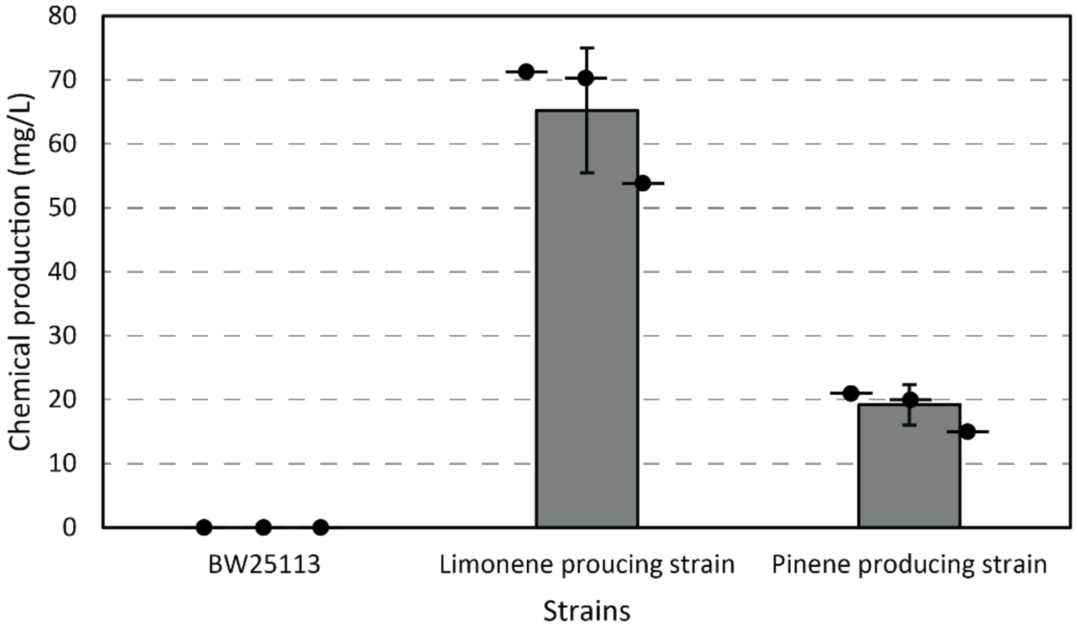

Supplementary Figure 12. **GC-MS of biofuel production levels in the whole cell culture.** Three independent experiments (black filled circles) for each strain were performed on the same day. The average and standard deviation of the data are shown as box plots and error bars, respectively.

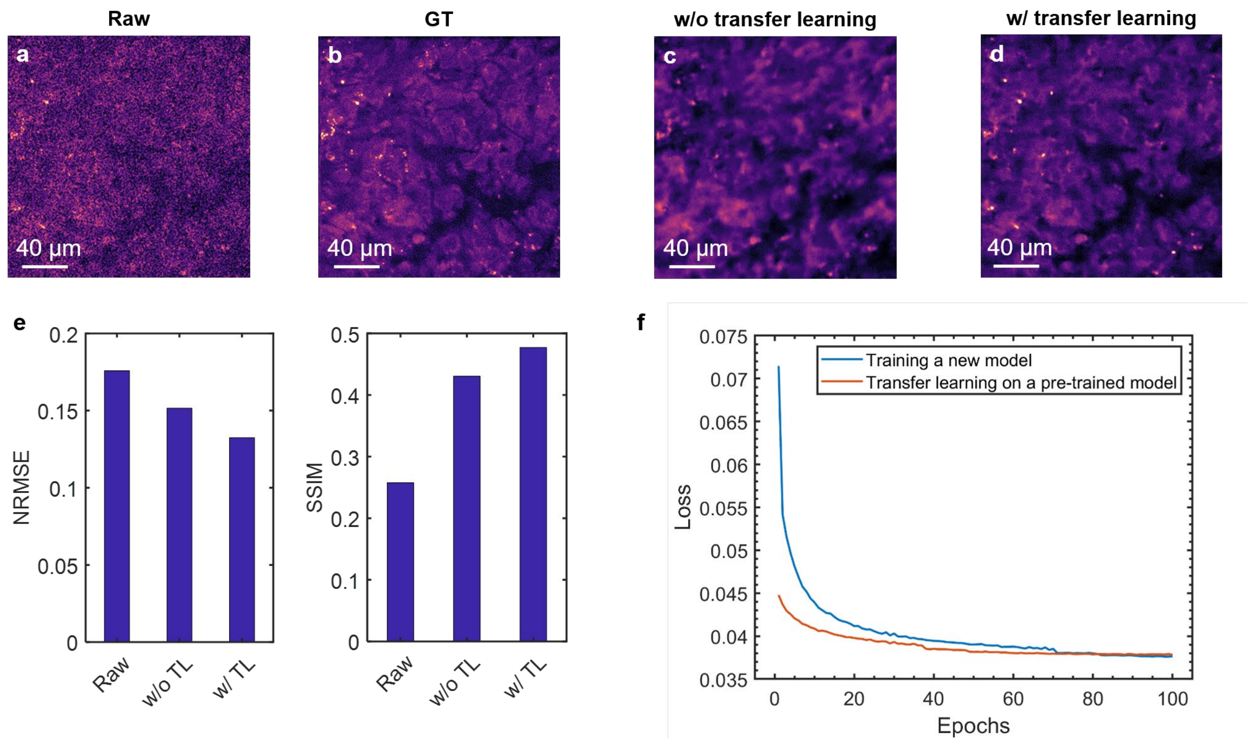

Supplementary Figure 13. **Transfer learning.** (a) Raw prostate cancer image (b) GT image by averaging 100 times. (c) Recovered prostate tissue image by applying a network trained on Mia PaCa-2 cells images. (d) Recovered prostate tissue image after transfer learning. (e) SSIM and NRMSE for the results shown in (a)-(d). The box represents the average value (n=13). (f) Training loss as a function of epochs by training a new model and transfer learning.

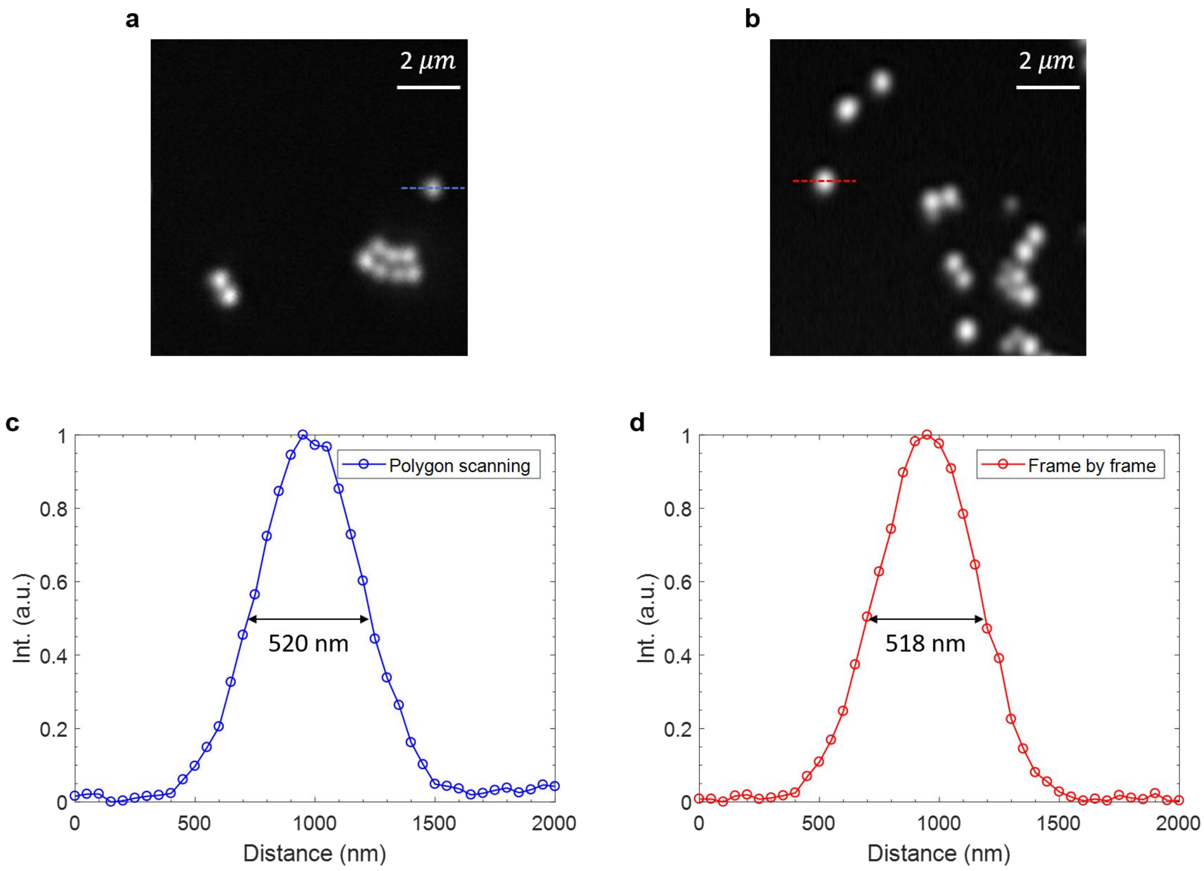

Supplementary Figure 14. **Spatial resolution characterization.** SRS (60X, 1.2 NA objective) at 2930 cm^{-1} of 500-nm PMMA beads by (a) Polygon-scanning and (b) Frame-by-frame setup. (c)-(d) Line intensity profiles of a single bead by polygon scanning and frame-by-frame. Independent experiments in 3 different FOVs were performed with similar results.

Video Legends

Supplementary video 1. Spectroscopic SRS imaging of live Mia PaCa-2 cells at 1650 cm^{-1} by raw acquisition.

Supplementary video 2. Spectroscopic SRS imaging of live Mia PaCa-2 cells at 1650 cm^{-1} after network recovery from raw acquisition.

Supplementary video 3. Protein chemical map of live Mia PaCa-2 cells after network recovery.

Supplementary video 4. Fatty acid chemical map of live Mia PaCa-2 cells after network recovery.

Supplementary video 5. Cholesterol chemical maps of live Mia PaCa-2 cells after network recovery.
