# Supplementary material for: Microsecond fingerprint stimulated Raman spectroscopic imaging by ultrafast tuning and spatial-spectral learning
Source: Nat Commun. 2021 May 24;12:3052. doi: 10.1038/s41467-021-23202-z (PMC8144602; doi:10.1038/s41467-021-23202-z)
Supplement: Supplementary file 3 — Description of Additional Supplementary Files [file 41467_2021_23202_MOESM3_ESM.docx]

**Description of Additional Supplementary Files**

**Supplementary video 1.** Spectroscopic SRS imaging of live Mia PaCa-2 cells at 1650 cm-1 by raw acquisition.

**Supplementary video 2**. Spectroscopic SRS imaging of live Mia PaCa-2 cells at 1650 cm-1 after network recovery from raw acquisition.

**Supplementary video 3.** Protein chemical map of live Mia PaCa-2 cells after network recovery.

**Supplementary video 4**. Fatty acid chemical map of live Mia PaCa-2 cells after network recovery.

**Supplementary video 5.** Cholesterol chemical maps of live Mia PaCa-2 cells after network recovery.
