# Supplementary material for: Microsecond fingerprint stimulated Raman spectroscopic imaging by ultrafast tuning and spatial-spectral learning
Source: Nat Commun. 2021 May 24;12:3052. doi: 10.1038/s41467-021-23202-z (PMC8144602; doi:10.1038/s41467-021-23202-z)
Supplement: Supplementary file 9 — Reporting Summary [file 41467_2021_23202_MOESM9_ESM.pdf]

nature research

Corresponding author(s): Ji-Xin Cheng

Last updated by author(s): Feb 22, 2021

Reporting Summary

Nature Research wishes to improve the reproducibility of the work that we publish. This form provides structure for consistency and transparency in reporting. For further information on Nature Research policies, see our Editorial Policies and the Editorial Policy Checklist.

Statistics

For all statistical analyses, confirm that the following items are present in the figure legend, table legend, main text, or Methods section.

n/a	Confirmed	
☐	☒	The exact sample size (n) for each experimental group/condition, given as a discrete number and unit of measurement
☐	☒	A statement on whether measurements were taken from distinct samples or whether the same sample was measured repeatedly
☒	☐	The statistical test(s) used AND whether they are one- or two-sided<br>*Only common tests should be described solely by name; describe more complex techniques in the Methods section.*
☒	☐	A description of all covariates tested
☒	☐	A description of any assumptions or corrections, such as tests of normality and adjustment for multiple comparisons
☐	☒	A full description of the statistical parameters including central tendency (e.g. means) or other basic estimates (e.g. regression coefficient) AND variation (e.g. standard deviation) or associated estimates of uncertainty (e.g. confidence intervals)
☒	☐	For null hypothesis testing, the test statistic (e.g. F, t, r) with confidence intervals, effect sizes, degrees of freedom and P value noted<br>*Give P values as exact values whenever suitable.*
☒	☐	For Bayesian analysis, information on the choice of priors and Markov chain Monte Carlo settings
☒	☐	For hierarchical and complex designs, identification of the appropriate level for tests and full reporting of outcomes
☒	☐	Estimates of effect sizes (e.g. Cohen's d, Pearson's r), indicating how they were calculated

Our web collection on statistics for biologists contains articles on many of the points above.

Software and code

Policy information about availability of computer code

Data collection	Data was collected by AlazarTech data acquisition card using lab-written Matlab code
Data analysis	Keras 2.2.4 (with Tensorflow as backend) was used to implement U-net for spectroscopic image denoising. LASSO hyperspectral image analysis was written in Matlab. All the code is available at https://github.com/buchenglab

For manuscripts utilizing custom algorithms or software that are central to the research but not yet described in published literature, software must be made available to editors and reviewers. We strongly encourage code deposition in a community repository (e.g. GitHub). See the Nature Research guidelines for submitting code & software for further information.

Data

Policy information about availability of data

All manuscripts must include a data availability statement. This statement should provide the following information, where applicable:

- Accession codes, unique identifiers, or web links for publicly available datasets
- A list of figures that have associated raw data
- A description of any restrictions on data availability

All the data related to the work is available upon reasonable request to the corresponding author. Example datasets for neural network training and spectral unmixing are available on the following website: https://github.com/buchenglab

Field-specific reporting

Please select the one below that is the best fit for your research. If you are not sure, read the appropriate sections before making your selection.

- [x] Life sciences
- [ ] Behavioural & social sciences
- [ ] Ecological, evolutionary & environmental sciences

For a reference copy of the document with all sections, see nature.com/documents/nr-reporting-summary-flat.pdf

Life sciences study design

All studies must disclose on these points even when the disclosure is negative.

Sample size	For each demonstration, we chose the sample size based on whether the trained network can restore the low-SNR images in the testing set to a high quality. The quality is quantitatively evaluated by calculating the SSIM and NRMSE between the ground truth image and the restored image as well as the chemical maps after spectral unmixing. Less than 20 pairs of 3D hyperspectral images were used for the training for MiaPaca2 and E. coli dataset. 50 images pairs were used for the training of the brain dataset.
Data exclusions	No data was excluded from analysis.
Replication	Data and code were available at https://github.com/buchenglab. The U-net can be trained from scratch using provided data. The model was trained several times and the results were reproducible.
Randomization	The training set was generated by randomly selecting a set of low/high SNR image pairs of cells or tissues in different conditions. A testing set was randomly selected which was not involved in the training.
Blinding	For each demonstration, the training and testing set was allocated randomly with no overlap. The neural network was solely trained and optimized based on the training set, the performance was evaluated by the testing set which was not observed by the network during the training phase.

Reporting for specific materials, systems and methods

We require information from authors about some types of materials, experimental systems and methods used in many studies. Here, indicate whether each material, system or method listed is relevant to your study. If you are not sure if a list item applies to your research, read the appropriate section before selecting a response.

Materials & experimental systems

n/a	Involved in the study	
x		Antibodies
	x	Eukaryotic cell lines
x		Palaeontology and archaeology
	x	Animals and other organisms
x		Human research participants
x		Clinical data
x		Dual use research of concern

Methods

n/a	Involved in the study	
x		ChIP-seq
x		Flow cytometry
x		MRI-based neuroimaging

Eukaryotic cell lines

Policy information about cell lines

Cell line source(s)	MIA PaCa-2 cells from ATCC
Authentication	Authenticated MIA PaCa-2 was obtained from ATCC more than 6 months ago. The cell line has not been authenticated after purchase
Mycoplasma contamination	Mycoplasma contamination test has not been tested after purchase.
Commonly misidentified lines (See ICLAC register)	None

Animals and other organisms

Policy information about studies involving animals; ARRIVE guidelines recommended for reporting animal research

Laboratory animals	Mouse, J:NU, male, age 21 days. Housing conditions are as follows: Light: dark/light 12/12 automatic timer. 325 lux (30-feet-candles) about 1 m above floor. Temperature: 21±3°C, accepted range 18-25°C (68-79°F). Humidity: Between 30-70%.

Wild animals	No wild animals were used in the study
Field-collected samples	No field-collected samples were used in the study
Ethics oversight	No ethical approval needed for this study since the tissue was collected from mouse that has been euthanized from other study.

Note that full information on the approval of the study protocol must also be provided in the manuscript.
